# Occurrence and characterisation of *Toxoplasma gondii* infection in free-living Eurasian beavers, *Castor fiber*, from southern England

**DOI:** 10.1038/s41598-025-26504-0

**Published:** 2025-11-25

**Authors:** Ambre Jacquart, Damer P. Blake, Simon Spiro, J. M. Jaramillo Ortiz, Rebecca Foskett, Sophie M. Common

**Affiliations:** 1 Department of Pathobiology and Population Sciences, Royal Veterinary College, Hawkshead Lane, North Mymms, Hatfield, AL9 7TA UK; 2 Institute of Zoology, Zoological Society of London, Regent’s Park, London, NW1 4RY UK

**Keywords:** *Toxoplasma gondii*, Eurasian beavers, Conservation translocation, Quantitative Polymerase Chain Reaction (qPCR), Genotyping, Histopathology, Genetic markers, Genotype, Infectious diseases, Neurological disorders, Conservation biology, Ecological epidemiology

## Abstract

*Toxoplasma gondii* is a globally distributed apicomplexan protozoan of zoonotic importance. Domestic cats (*Felis catus*) and related felids constitute its sole definitive hosts, while numerous warm-blooded species serve as intermediate hosts*.* Infection outcomes for intermediate hosts range from latent toxoplasmosis to behaviour alteration and fatal systemic disease. Oocysts, the lifecycle environmental stage, are often washed into watercourses, making river-dwelling species like Eurasian beavers (*Castor fiber*) particularly at risk. To investigate the presence and impact of *T. gondii* on beavers in the United Kingdom, archived brain, heart, and liver from 23 beavers necropsied between 2021 and 2023 were screened for *T. gondii* DNA by nested- and quantitative PCR. Positive samples were genotyped using a panel of six published PCR-Restriction Fragment Length Polymorphism (PCR-RFLP) assays and amplicon sequencing. Sequences were aligned with those reported previously from UK and other wildlife species to assess the occurrence of shared genotypes. *Toxoplasma gondii* DNA was detected in 19/23 (83%) Eurasian beavers; all consistent with PCR-RFLP ToxoDB#3 with limited genetic diversity, concurring with findings from previous surveillance of UK wildlife species. Following histopathological screening non-suppurative toxoplasmosis was characterised in 3/6 tissues selected from two animals. These results demonstrate a high occurrence of *T. gondii* infection in UK beavers

**Supplementary Information:**

The online version contains supplementary material available at 10.1038/s41598-025-26504-0.

## Introduction

Eurasian beavers (*Castor fiber*) have been released into outdoor fenced enclosures at over 30 locations in southern England, however escapes from unregulated enclosures and unauthorised releases, including from populations of unknown origin, have led to the establishment of a fragmented metapopulation structure with free-living beavers now present across seven separate locations^1^. In response to this uncoordinated reintroduction framework, the Disease Risk Analysis and Health Surveillance (DRAHS) group from the Zoological Society of London (ZSL), in partnership with Natural England (NE), implemented a passive disease surveillance program starting in 2021. The project aims to improve understanding of the health, disease and genetic status of these populations ahead of possible future officially licensed reintroductions into England. A Disease Risk Analysis (DRA) was produced for the reintroduction which qualitatively assessed relevant infectious and non-infectious hazards, and has been used to guide disease surveillance work^2^. Since 2021, post-mortem examinations (PMEs) have been performed on beaver carcasses retrieved and submitted from free-living and captive populations in England, including targeted surveillance for the zoonotic protozoan parasite *Toxoplasma gondii.*

*Toxoplasma gondii* is a globally distributed apicomplexan protozoan of major zoonotic significance, capable of causing toxoplasmosis in a wide range of warm-blooded intermediate hosts with severe clinical outcomes in immunocompromised individuals and congenitally infected fetuses^3^. The parasite undergoes sexual reproduction exclusively within the intestinal epithelium of felids, its definitive hosts, leading to the excretion of environmentally resistant oocysts in feces, which serve as a primary source of transmission^3^. Intermediate hosts are then exposed and infected through ingestion of sporulated oocysts, which convert into tachyzoites (causing the acute stage of disease) and eventually bradyzoite cysts in tissues (latent toxoplasmosis); transmission can also occur by ingestion of cysts in tissues^4^. There is evidence that beavers can act as intermediate hosts for *T. gondii*, and both infection and disease have been reported in captive and free-living beavers throughout Europe^5–7^. Infection may have deleterious conservation implications for beavers due its potential negative impact on post-release survival^2^. Stress, such as that associated with the translocation process but also experienced naturally in the wild (e.g. competition, concurrent disease or injury, resource scarcity)^8,9^, can lead to disease recrudescence as a result of immunomodulation^10^. *Toxoplasma gondii* tissue cysts have been detected in various organs of intermediate hosts, including the brain, skeletal muscle, heart, kidneys and liver^3^, and the predilection of *T. gondii* for neural tissue has been associated with behavioural alterations in rodents including neophobia and decreased reaction time, suggesting that exposure to *T. gondii* may predispose beavers to hazards such as road traffic collisions (RTCs) or predation^2,11^.

Exposure of beavers to *T. gondii* may be exacerbated by the parasite’s high resilience in the environment. Environmentally resistant oocysts can disseminate via environmental matrices including fresh water^12^, and may be transmitted from terrestrial to aquatic habitats via sewage systems or stormwater drainage and freshwater runoff^13–15^. Beavers may be particularly at risk due to their behavioural ecology – they live in stagnant pools as a result of their damming activity, which can lead to oocyst accumulation in their aquatic environment^16^. Whilst Eurasian beavers are unlikely to present a substantial source of infection to other host species, *T. gondii* prevalence amongst free-living beavers may be exacerbated by their proximity to human-populated areas due to higher definitive feline host density, and hence higher environmental oocyst contamination^5,17^. Surveillance of beaver populations can serve as sentinels of parasite occurrence.

*Toxoplasma gondii* global population structure is largely constituted around three main lineages (Types I, II, and III), with this genetic stability over time and space attributed to limited sexual recombination, solely occurring in felids, and frequent asexual replication in intermediate hosts^4^. Hybrid genotypes do occur but are rare in many regions, with genetic diversity and evidence of recombination more frequently observed in South America and parts of Africa, likely due to higher host diversity and co-infection rates^4^. A study aimed at determining the occurrence and genetic diversity of *T. gondii* within wild British carnivores detected the presence of all three archetypal *T. gondii* clonal lineages (I, II, and III), including the Eurasian badger (*Meles meles*), ferret (*Mustela furo*), red fox (*Vulpes vulpes*), American mink (*Neovison vison*), European polecat (*Mustela putorius*) and stoat (*Mustela erminea*)^18^. However, more recent evidence has suggested that *T. gondii* population structure can be complex in some ecosystems, with atypical strains presenting unique polymorphisms^19^. Associations between *T. gondii* genotype and pathogenesis have been scarce in wildlife populations, highlighting the value of molecular typing and comparison with histological findings when infection is detected^7^.

This study aimed to investigate the occurrence of *T. gondii* in Eurasian beaver populations from southern England, including both wild-living individuals and those living in large, naturalistic, and largely unmanaged fenced enclosures, to characterize the *T. gondii* genotypes, and assess the pathological relevance of toxoplasmosis in this species, with the aim to better understand *T. gondii* epidemiology in Eurasian beavers in England.

## Results

### *Toxoplasma gondii* occurrence in Eurasian beavers

A highly sensitive and widely used nested PCR assay targeting a repetitive 529 bp DNA fragment in *T. gondii*^20^ was performed on 19 brain, 23 heart and 23 liver samples from 20 free-living and 3 captive beavers. *Toxoplasma gondii* DNA was detected in at least one tissue from 19/23 (83%) beavers, including in all 3/3 captive beavers (80% in free-living and 100% in captive beavers). *Toxoplasma gondii* DNA was most frequently detected in brain samples (14/19), followed by liver (12/23) and heart (9/23). All three organs tested positive in six beavers (Table 1). No template controls were included in all assays to control for DNA contamination.

**Table 1:** Detection and quantification of *Toxoplasma gondii* genomic DNA in tissues collected from Eurasian beavers (*Castor fiber*) at post-mortem.

Beaver ID	Habitat	qPCR ratio (SQ *T. gondii* genome copies/SQ beaver genome copies)			Carcass autolysis
		Brain	Heart	Liver	
*XT585-21*	Free-living	NA	–	0.00005	Mild
*XT607-21*	Free-living	–	0.00013	0.00003	Severe
*XT619-21*	Free-living	**0.00883**	**0.00225**	0.00003	Mild
*XT034-22*	Free-living	NA	–	0.00002	Moderate
*XT145-22*	Free-living	–	–	0.00003	Mild
*XT216-22*	Free-living	NA	**0.03928**	–	Severe
*XT415-22*	Free-living	**0.00158**	0.00013	0.00006	Moderate
*XT583-22*	Free-living	0.00003	0.00005	0.00005	Moderate
*XT584-22*	Free-living	0.00051	–	0.00027	Moderate
*XT040-23*	Captive	0.00011	0.00008	–	Mild
*XT053-23*	Free-living	**0.02768**	–	**0.00862**	Moderate
*XT145-23*	Free-living	–	–	0.00001	Moderate
*XT157-23*	Free-living	**0.00517**	0.00028	–	Mild
*XT189-23*	Free-living	**0.02444**	–	–	Severe
*XT451-23*	Captive	0.00037	0.00050	0.00002	Mild
*XT637-23*	Free-living	**0.00232**	–	–	Severe
*XT705-23*	Free-living	**0.11440**	–	–	Severe
*XT819-23*	Free-living	0.00009	0.00015	0.00021	Severe
*XT820-23*	Captive	**0.00168**	–	–	Mild

Each sample was first tested by nested PCR targeting a 529 bp repeat element within the *T. gondii* genome; positive samples were subsequently quantified by qPCR to assess suitability for genotyping, calculated as the number of *T. gondii* genome copies/ beaver genome copy. Samples represented by ratios > 0.001 were used for further genetic characterisation, highlighted in bold. Samples negative by nested PCR are indicated by “–” and samples that were not available to test are indicated by “NA”.

The number of *T. gondii* genome copies in positive samples was then enumerated by qPCR. All nested PCR positive samples had detectable levels of *T. gondii* genomic DNA by qPCR targeting the *T. gondii* 529 bp repeat (Ct range 20.38–39.67, threshold considered positive ≤ 38.00), with between 0.11440 and 0.00001 parasite genomes detected per host genome (assuming ~ 250 repeat copies per parasite genome; Table 1). Samples presenting parasite:host ratio values over 0.001 were selected for PCR-Restriction Fragment Length Polymorphism (PCR-RFLP). Several severely autolysed samples yielded very high ratio values and were not progressed to histologic examination.

### PCR-RFLP characterisation of *T. gondii* genotypes

Eighteen of 19 beavers found to be positive for *T. gondii* genomic DNA were represented by at least one organ sample with more than 0.001 T*. gondii* genome copies per host genome and were retained for PCR-RFLP characterization. A panel of six PCR-RFLP assays was applied^21^. All (18/18) samples were genotyped at the SAG3 locus, but incomplete amplification and/or genotyping was observed at other markers (GRA6: 12/18, 5′SAG2: 11/18, 3′SAG2: 12/18, BTUB: 5/18, APICO: 3/18; Table 2). Two samples were genotyped at all six loci, presenting a primarily type II profile corresponding to the lineage variant assigned PCR-RFLP Genotype Number 3 (ToxoDB#3, samples XT415-22 and XT053-23; Table 2). Of 16 incomplete sample profiles, all were consistent with the same ToxoDB#3 annotation. All markers presented as type II with the exception of APICO, which was annotated as type I in all successfully typed examples. Comparative analysis of the SAG3 PCR-RFLP amplicon sequences using BLASTn against the GenBank core nucleotide database identified 100% sequence identity and coverage to the archetypical ME49 type II lineage for 13 of 18 samples (accession numbers PV059145-PV059157). The other 5 SAG3-typed sequences obtained solely from free-living beavers were genetically distinct from other sequences in this study and currently available in GenBank, including single nucleotide polymorphisms further distinguishing the subtype (accession numbers PV059158-PV059162).

**Table 2:** *Toxoplasma gondii* PCR-RFLP genotypes from Eurasian beavers using six loci.

Beaver ID	SAG3	5′ SAG2	3′ SAG2	GRA6	BTUB	APICO	ToxoDB genotype*
*XT585-21*	II	I/II	II	II	–	–	[3]
*XT607-21*	II	–	II	–	–	–	[3]
*XT619-21*	II	I/II	II	II	–	–	[3]
*XT034-22*	II	–	II	x	–	–	[3]
*XT216-22*	II	–	–	–	–	–	[3]
*XT415-22*	II	I/II	II	II	II	I	**3**
*XT583-22*	II	–	II	II	II	–	[3]
*XT584-22*	II	I/II	II	II	–	–	[3]
*XT040-23*	II	I/II	II	II	x	–	[3]
*XT053-23*	II	I/II	II	II	II	I	**3**
*XT145-23*	II	–	II	II	–	–	[3]
*XT157-23*	II	I/II	II	x	x	–	[3]
*XT189-23*	II	I/II	–	II	x	–	[3]
*XT451-23*	II	I/II	x	II	–	I	[3]
*XT637-23*	II	–	–	–	–	–	[3]
*XT705-23*	II	–	–	–	–	–	[3]
*XT819-23*	II	I/II	II	II	II	–	[3]
*XT820-23*	II	I/II	–	II	II	–	[3]

– = failed PCR-RFLP amplification. x = failed sequencing. *RFLP Genotype Number assigned following ToxoDB numbering: full profiles shown in bold, incomplete but suggestive shown in square brackets.

### Phylogenies between Eurasian beavers and UK wildlife species

**Fig. 1:**
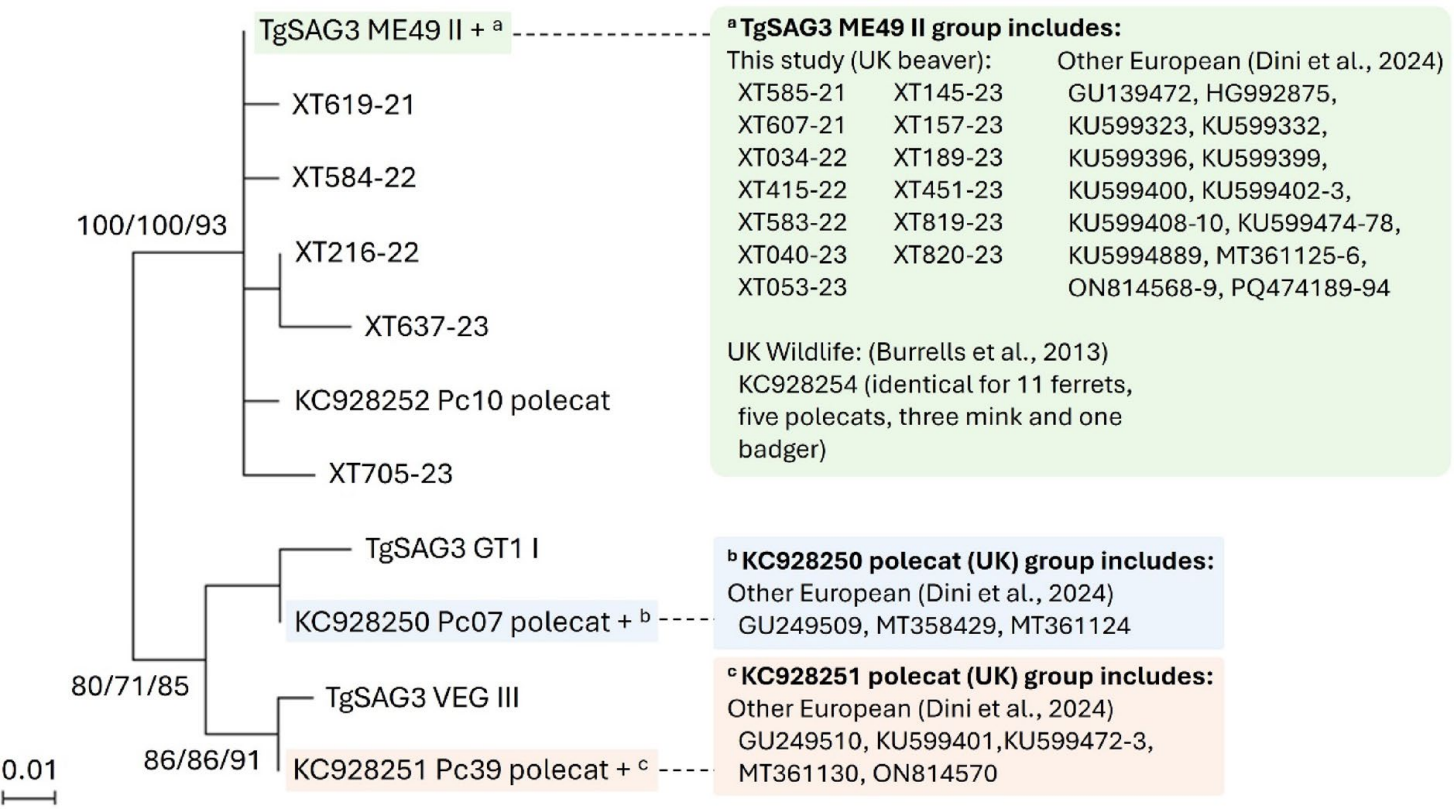
Optimal Maximum Likelihood phylogeny for *Toxoplasma gondii* PCR-RFLP SAG3 sequences from 18 beavers, 9 polecats, 11 ferrets, 3 mink, and 1 badger from the UK, compared to 38 published European sequences. Generated using an alignment of 145 base pairs with reference sequences from non-beaver UK wildlife and other European sequences indicated by accession numbers. Sequences from the reference GT1, ME49 and VEG strains included as representatives of the I, II and III archetypes accessed from ToxoDB. The sequences denoted with superscript letters represent clusters of identical sequences. Support for each node is presented, indicating outcomes from Maximum Likelihood (ML)/Neighbor-Joining (NJ)/ Unweighted Pair Group Method with Arithmetic Mean (UPMGA) methods. Beaver sequences available under the accession numbers PV059145-PV059162.

Phylogenetic analysis was carried out using a 145 bp fragment of the *T. gondii* SAG3 PCR-RFLP amplicon, the most widely represented sequence in the dataset. Published reference sequences representing the SAG3 amplicon from the three archetypal *T. gondii* lineages were aligned with SAG3 sequences from 18 infected beavers (this study) and 24 published sequences from other UK wild animals (11 ferrets, 9 polecats, three mink and one badger, accessed from GenBank; Burrells et al*.* (2013) for comparison). Analysis was supplemented with a panel of 38 published *T. gondii* SAG3 sequences derived from humans, farmed and wild animals sampled across Europe^18,22^. Results confirmed the type II genotype for all beaver samples, with 13 found to be identical to ME49, 20 sequences from wild UK animals, and 29/38 published sequences from samples collected in Europe (Fig. 1). SAG3 sequences most closely comparable to the archetypical lineages I and III were previously described in two (accession number KC928250) and one (accession number KC928251) UK polecats, and three or six European samples, but were not detected here. Five novel type II *T. gondii* SAG3 sequences were also amplified here from beavers, with no previous identical matches from the UK or in GenBank.

### Histopathological hallmarks of toxoplasmosis

**Fig. 2:**
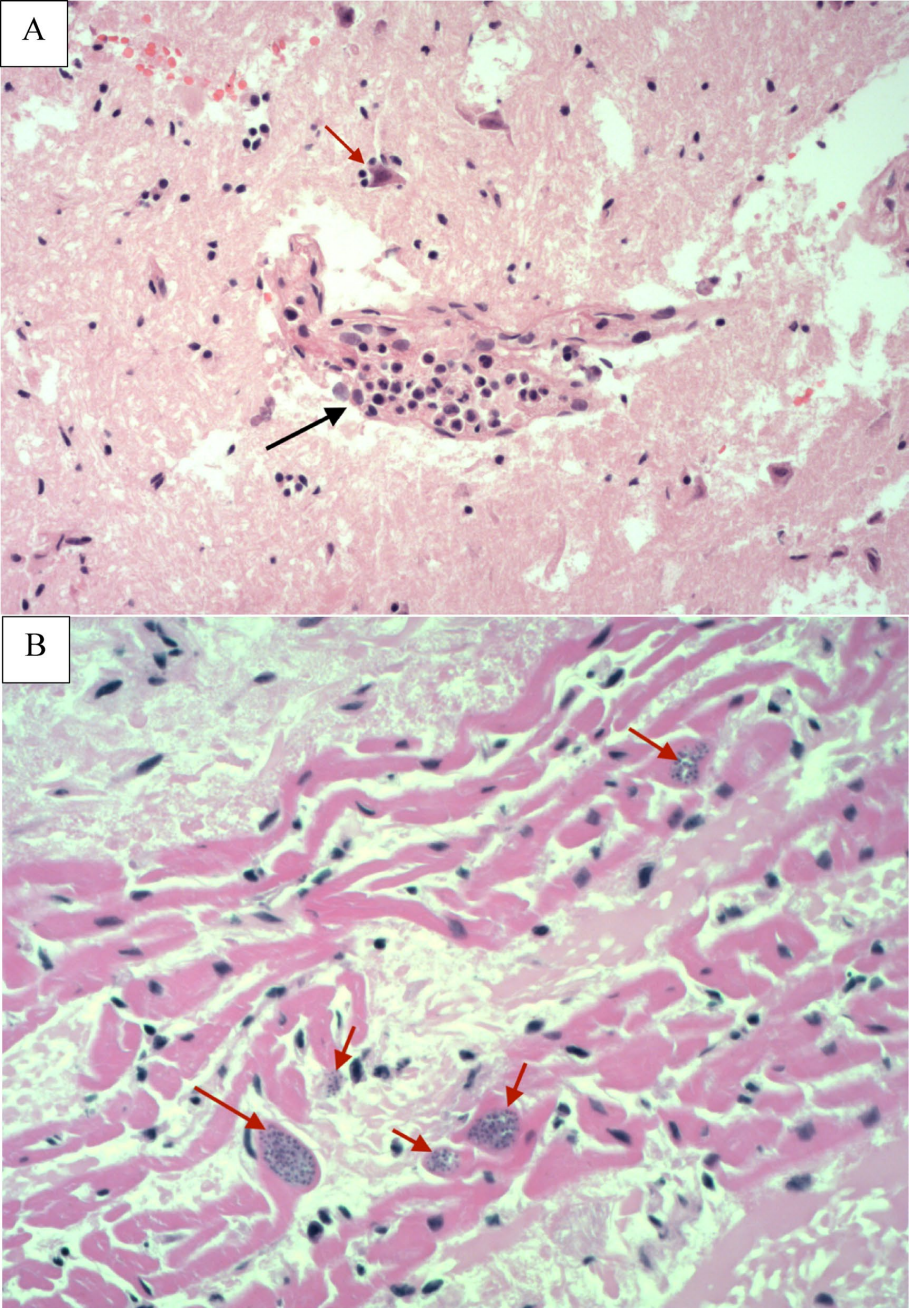
Histopathologic hallmarks of toxoplasmosis infection in Eurasian beaver XT053-23 (H&E stain, 20 × objective). (**A**) Perivascular cuffing (red arrow) adjacent to neuron with signs of satellitosis (black arrow). (**B**) Intracytoplasmic protozoal cysts within cardiomyocytes (red arrow). Identification supported by commercial immunohistochemistry.

Tissues were selected for histopathological examination where formalin fixed tissues were available, autolysis had been scored as limited at PME and qPCR results for at least one organ were above 0.001 T*. gondii* genomes per host genome. Two animals (XT053-23 and XT415-22) most closely met the inclusion criteria, although autolysis had been scored as moderate for both (Table 1). Protozoal tissue cysts consistent with *T. gondii* were seen in the heart of XT053-23 and non-suppurative encephalitis consistent with toxoplasmosis was seen in the brain. Tissue cysts were not observed in XT415-22. Hallmarks of toxoplasmosis infection were evidenced in 3/6 tissues examined, specifically in XT053-23 brain and heart tissues as well as in XT415-22 brain tissue; more significant encephalitis signs (mononuclear cell aggregation, perivascular cuffing, gliosis) were characterised for XT053-23 (Fig. 2A). A localized cluster of bradyzoite cysts within cardiac muscle fibers was also present in XT053-23 heart tissues (Fig. 2B), despite yielding a negative PCR (PCR positive in brain and lung; Table 1). Immunohistochemical staining confirmed the presence of *T. gondii* in both the myocardium and brain of XT053-23 but, in line with the absence of tissue cysts detectable by light microscopy, did not reveal parasite stages within the brain histological section of XT415-22.

## Discussion

This study reports the highest occurrence of *T. gondii* to date in a population of Eurasian beavers (83%; n = 19/23). Previous research on parasite detection in beaver carcasses retrieved from the wild described infection rates of 56% (23/41) in Switzerland^5^ and 6% in the Netherlands^6^, although different screening methods (Indirect ELISA and MS-qPCR) used in the latter study mean that the results are not directly comparable. Our findings suggest high sensitivity of both the nested PCR and qPCR assays, regardless of parasite DNA occurrence in tissues and carcass preservation. As *T. gondii* tissue cysts can appear as early as 10 days post-infection and persist long term in tissues^23^, successful parasite detection by nested PCR supports use of molecular diagnostics in surveillance^24^. The addition of qPCR ratios constituted an effective indicator of infection intensity when tissue autolysis was low, with high analytical sensitivity of this method previously described in the literature^25^. Here, qPCR was adopted after nested PCR to assess suitability for PCR-RFLP genotyping rather than as a primary diagnostic. The highest occurrence of *T. gondii* DNA was detected in tissue samples considered to be severely autolyzed, suggesting faster degeneration of beaver DNA compared to *T. gondii* DNA, although the low sample size precludes a firm conclusion^26^. *Toxoplasma gondii* was most detected and displayed higher parasite DNA levels in brain samples compared with the corresponding heart and liver samples, despite autolysing more rapidly due their high water content^27^. These findings support the hypothesis that latent *Toxoplasma* infection is common in beavers and that brain should be prioritised for future molecular screening for *T. gondii* infection, consistent with previous research^5,24^.

There are several possible reasons for the increased *T. gondii* occurrence detected in the current study compared to that reported in beavers from other areas of Europe. Free-living beaver populations in Southern England have resulted from unreported translocations, and may, therefore, have originated from European countries where *T. gondii* is highly prevalent. Alternatively, individuals may have originated from captive settings or escaped from enclosed environments^1,4^. Elevated occurrence of *T. gondii* in captivity, relative to natural conditions, has been documented across multiple rodent species: this pattern is consistent with the findings of the present study, in which *T. gondii* was detected in all captive beavers examined. However, the sample size was limited to three individuals, and thus should be interpreted with caution^2^. A higher density of domestic felids in or near beaver habitats as well as anthropogenic activities such as irresponsible cat litter disposal or inadequate sewage/filtration systems may influence acquired infection rates in beavers by enhancing their risk exposure to environmental *T. gondii* oocysts^5,15,28^. The risk of selection bias associated with passive surveillance efforts constitutes a limitation for disease prevalence estimations at the population level, but is commonly used for wild animal populations^29^.

Semi-aquatic species may exhibit a higher parasite prevalence due to their exposure to oocysts contained in both terrestrial and aquatic environments*.* Lower *T. gondii* infection rates have been reported in British terrestrial mammals (varying from 6.0 to 44.4% depending on the species) when compared with beavers in the current study^18^. In aquatic environments, the concentration of *T. gondii* oocysts is inversely proportional to distance from the shore, due to factors like freshwater run off^15^. Thus, parasite exposure is likely to be higher for in-land freshwater aquatic mammals compared with in- or off-shore marine species^30,31^. Beavers may, therefore, constitute suitable sentinels for assessment of oocyst contamination levels in freshwater ecosystems, similar to other inland species of higher trophic levels like sea otters^15^.

All *T. gondii* PCR-RFLP types detected in this study were most closely comparable to the type II archetypical lineage with low diversity in all sequences generated. Previous studies with UK wildlife have also found type II to be most common, although types I and III have been detected in polecats, where a carnivorous lifestyle and a large prey range may enhance exposure to a wider range of *T. gondii* genotypes^18^. Here, a *T. gondii* PCR-RFLP Genotype Number 3 (ToxoDB#3) profile corresponding to primarily type II variants was identified in all samples tested, with single nucleotide polymorphisms further distinguishing subtypes^32^. Parasites related to reference type II strains such as ME49 are most prevalent in Europe^33^, and the ToxoDB#3 genotype has been identified in numerous Eurasian beavers, lynx and voles from Switzerland, as well as livestock across Europe, implying a low spatial variation of *T. gondii* genetic diversity^5,22,34^. Phylogenetic comparison of a short fragment of the SAG3 PCR-RFLP amplicon supported the suggestion of limited genetic variation, although six distinct genotypes were detected, five of which were new to GenBank. The short sequence length available for analysis prevented more robust phylogenetic comparison. Type II *T. gondii* strains have been found to cause subclinical disease in rodents^35^. However, mice infected with this strain have previously showed neurological deficits^36^. Here, histopathological assessment of two beavers identified hallmarks of toxoplasmosis. The same genotype (nuclear type II, APICO I) and histopathological findings as in beavers XT053-23 and XT415-22 have previously been described in a free-living beaver suffering from fatal disseminative toxoplasmosis^7^. *Ante-mortem* neurologic signs of reduced consciousness, uncoordinated swimming and sustained periods of immobility prior to death were observed in a captive harbour porpoise infected with this type II, APICO I variant. It is possible that similar behavioural alterations may occur in Eurasian beavers infected with *T. gondii*, leaving them at increased risk of exposure to threats such as road traffic collisions, the most frequent cause of death for Eurasian beavers identified by a long-term analysis in eastern Germany^2,37^. The statistical power in the current study was insufficient to assess potential associations between *T. gondii* infection and circumstances of death, but this may be explored in future work when the sample size is increased.

Beaver translocations alone are unlikely to directly influence the spatiotemporal distribution of *T. gondii* genotypes and local variations in genetic population structure within Southern England, but the sudden appearance of atypical virulent *T. gondii* isolates has previously been documented in aquatic and marine wildlife^12,38^. Therefore, genotyping efforts should be included in *T. gondii* surveillance and should target the most sensitive and informative markers. For example, a study on Swiss beavers characterised a mutation in the SAG3 gene which codes for a tachyzoite surface protein associated with target cell attachment and immune modulation^39^. This genetic marker may have differentiated Swiss genotypes with increased pathogenicity since inflammatory changes were observed in 26/40 brain histological specimens^5^. Here, genotyping success varied between samples and markers, likely influenced by variation in tissue autolysis and parasite DNA concentrations. Locus-specific amplicon length may have played a role in marker sensitivity; amplification efficiency decreases as amplicon length increases and the risk of allelic drop-out is higher when DNA degradation is elevated^40^. However, the finding of a single, apparently conserved PCR-RFLP genotype in all samples precluded any inference between genotypes identified and their potential pathogenesis.

The pathological relevance of toxoplasmosis in Eurasian beavers described by others was supported by our study, although the sample size was very small^5^. One of two animals assessed had non-suppurative encephalitis consistent with protozoal infection and, while *T. gondii* organisms were not directly observed in the affected tissue, they were detected by molecular assay. Cysts consistent with *T. gondii* were observed in the heart of this animal, but PCR of this tissue was negative. This discrepancy could indicate a lower parasite detection sensitivity by molecular assay than previously described or the identification of protozoan cysts other than *T. gondii* in the beaver’s tissues upon polyparasitism infection^41,42^. Immuno-histochemistry was successfully employed to confirm *T. gondii* identity in the heart from this animal, although application to a second individual positive for *T. gondii* DNA but negative by microscopy was unsuccessful. Routine application of immuno-histochemistry approaches in future diagnostic efforts would allow differentiation from concomitant infections, for example with *Sarcocystidae* parasites^2,43^**,** but also potentially characterise the presence of tachyzoites upon acute toxoplasmosis which can be challenging by H&E staining alone. Nonetheless, the occurrence of rare “hot spots” of parasite replication by multiple bradyzoites released locally from a tissue cyst (concurrent with the histological observations in this beaver) has been described in the literature and may explain this discrepancy: tissue cysts are heterogeneously distributed in the heart and might not have been present in the tested sample^44^. Interestingly, the occurrence of protozoan cysts in cardiomyocytes was associated with left ventricular myocardial thickening in beaver XT053-23. The same gross pathological change was observed in two domestic cats presenting with signs of acute myocarditis secondary to toxoplasmosis, although both pathologies have a wide range of clinical presentations hence their association can only be presumptive^45^. Application of a grading system for histopathological changes would allow for a more ‘quantitative’ assessment of the pathological implication of *T. gondii* infection with a bigger study population size^42^; here, lack of statistical power prevented identification of potential associations between parasite occurrence and histopathological changes.

*Toxoplasma gondii* surveillance in Eurasian beavers is valuable due to reintroduction considerations and their role as sentinels of freshwater contamination with oocysts. Translocation of beavers could lead to acute toxoplasmosis resulting from chronic disease recrudescence under stressful conditions or secondary to an injury or infection but also constitute a potential hazard due to the association of latent toxoplasmosis with neurological disorders challenging beaver survival in the wild. Thus, the high parasite occurrence characterized in our study should be considered in the light of future reintroduction efforts.

## Materials and methods

### Study area and population

Twenty-three beaver carcasses, necropsied between May 2021 and November 2023 as part of a health surveillance program carried out by the Zoological Society of London, were selected for inclusion in the current study. The study population consisted of 20 males, two females, and one beaver of unknown sex. Eleven beavers were classified as adults, seven as sub-adults and five as kits on the basis of morphometric measurements^46^. Three carcasses originated from captive facilities, whilst the rest of the study population were free-living beavers retrieved from the wild; all beavers were found in Southern England (Table 1).

### Post-mortem tissue sampling

Upon post-mortem examination (PME), approximately 1 g of brain, heart and liver tissue were collected from each beaver and placed into either plastic or glass containers, then stored at − 20 °C or − 80 °C prior to processing. Carcass autolysis was qualitatively assessed as mild, moderate or severe by a board-certified veterinary pathologist at the time of PME.

### Detection and quantification of *T. gondii* DNA occurrence

Total genomic DNA extraction was carried out using a DNeasy Blood and Tissue Kit (Qiagen, Crawley, UK) following the manufacturer’s instructions with the following modifications. Tissue samples of approximately 1 g were thawed and homogenized in 1000 µL buffer ATL using a Qiagen TissueRuptor until disruption was complete. A sub-sample of 50 µl was transferred to a new 1.5 ml microtube and 20 µl Proteinase K was added. Microtubes were mixed by vortexing and incubated at 56 °C for 3 h with intermittent vortexing every hour. After vortexing for 15 s, the DNA extraction protocol was completed using DNeasy spin columns as recommended by the manufacturer and the purified total genomic DNA was eluted in 200 µl Buffer AE.

The presence of *T. gondii* genomic DNA was detected using a nested PCR assay targeting a 529 bp repeat sequence within the *T. gondii* genome as described by Su et al.^32^ with both amplifications optimized in a 25 μl reaction volume containing 12.5 µl 2 × MyTaq Mix (Bioline, London, UK) and 11.3 µl molecular grade water. The primary amplification mix contained 0.2 µl of 100 µM primers NF1 (5′-TGACTCGGGCCCAGCTGCGT-3′) and NR1 (5′-CTCCTCCCTTCGTCCAAGCCTCC-3′), synthesised by Merck, Haverhill, UK. The secondary amplification mix contained 0.2 µl of 100 µM primers NF2 (5′-AGGGACAGAAGTCGAAGGGG-3′) and NR2 (5′-GCAGCCAAGCCGGAAACATC-3′). A volume of 1 µl DNA extraction product was included in the first nested PCR assay; and 1 µl of the first amplification product was included in the 2nd round nested PCR assay. PCR cycles for both rounds of nested amplification were 94 °C for 0.5 min, followed by 35 cycles of 94 °C for 0.5 min, 58 °C for 0.5 min and 72 °C for 0.5 min, with a final extension step of 72 °C for 10 min using a G-Storm thermal cycler (VWR, Lutterworth, UK). Molecular grade water (Sigma, Welwyn Garden City, UK) was used as template in place of DNA as the no template control. Expected amplicon sizes were 419 bp and 164 bp for the first and second round nested PCRs, respectively.

Nested PCR amplification products were resolved by agarose gel electrophoresis through a 1% (w/v) UltraPure agarose gel (Thermofisher Scientific, Milton Keynes, UK) prepared using 50 ml 1 × TBE (Tris–Borate EDTA; Invitrogen, Paisley, UK) buffer and stained using 5 μl SafeView (0.01% v/v; NBS Biologicals, UK). Well loading volumes of 7 µl PCR products were mixed with 1 µl TriTrack loading dye (Thermofisher Scientific). A DNA ladder (GeneRuler 1 kb + ; Thermofisher Scientific) was included to establish amplicon size. Gel results were visualized using a Syngene U-Genius imaging system (Syngene, Cambridge, UK).

### Quantitative Polymerase Chain Reaction (qPCR)

Quantitative PCR (qPCR) was used to screen samples found to contain *T. gondii* DNA by nested PCR to identify samples with sufficient *T. gondii* DNA for PCR-RFLP. *Toxoplasma gondii* genome number was quantified by targeting a 188 bp T*. gondii* sequence within the 529 repeat-element using the forward primer Tox-9F (5′-AGGAGAGATATCAGGACTGTAG-3′) and the reverse primer Tox-11R (5′-GCGTCGTCTCGTCTAGATCG-3′) as described in Bachand et al*.*^13^; normalised using primers targeting the host *Pelo* gene (Pelo_F 5′-TCCACTATCCGCAAGGTTCAG-3′, Pelo_R 5′-GGCTTGGGAGTCAAAGTCGAT-3′)^47^. Quantitative PCR amplification was performed in 96 well plates using the Bio-Rad CFX96 Real time detection system (Bio-Rad laboratories, USA) with SsoFast EvaGreen Supermix (Bio-Rad, USA) in 20 μl reaction volumes per well. Each reaction consisted of 7 μl of EvaGreen Supermix, 1 μl each forward and reverse primer (100 μM), 10 μl molecular grade water (Sigma) and l μl template DNA. Cycling conditions for the reactions included initial denaturation at 95 °C for 5 min, followed by 40 cycles of denaturation at 95 °C for 10 s and annealing/extension at 60 °C for 20 s. Melt curves were determined at 65–95 °C. Each sample was tested in triplicate, with host/parasite genome-specific standard dilution series run on each plate using a single well per concentration and molecular grade water as the non-template (negative) control. Quantification cycle threshold value (Ct) was used to assess the *T. gondii* status of each sample following normalisation against host genome copy number. Following optimisation of the assay (data not shown), samples with more than 0.001 T*. gondii* genomes (assuming ~ 250 copies of the 529 repeat target per genome) per host genome were subsequently selected for histopathology. A Ct value < 38.00 was considered to be positive.

### PCR-RFLP multi-locus genome typing

Nested PCR positive samples were genotyped using six published PCR-Restriction Fragment Length Polymorphism (PCR-RFLP) assays targeting the loci 5′SAG2, 3′SAG2, SAG3, B-BTUB, GRA6, and APICO^32^. Amplifications were optimized in 25 µl using the same solution volumes as for the nested-PCR assay but including 2 µl of DNA template. Reactions were incubated at 94 °C for 4 min, followed by 30 cycles of denaturing for 30 s at 94 °C, annealing for 30 s at 55 °C, and extension for 2 min at 72 °C. The final cycle was followed by an extension step of 10 min at 72 °C. Two µl of each final PCR product was then used as template DNA in the secondary PCR for 35 cycles with an annealing temperature of 60 °C for 30 s. The forward and reverse primers and associated fragment length for each marker are displayed in the Supplementary Materials (Supplementary Table S1). Amplicon size was confirmed by agarose gel electrophoresis and purified PCR amplicons were sent for Sanger sequencing (Source Bioscience, Nottingham, UK) using the inner nested PCR primers employed in their amplification. Sequencing results were curated and analysed using CLC Main Workbench (version 8.1.3, Qiagen) and PCR-RFLP genotypes were identified by presence or absence of the RFLP restriction endonuclease recognition site (Supplementary Table S1).

### Phylogenetic analysis

A 166 bp fragment of the SAG3 PCR-RFLP amplicon achieved widest representation within the dataset and was used for phylogenetic analysis. Published sequences representing the SAG3 amplicon sequence from three clonal archetypal *T. gondii* lineages (used as reference sequences) were accessed using ToxoDB (https://toxodb.org/toxo/app), with GT1– type I, ME49– type II and VEG– type III. Reference sequences were aligned with the SAG3 sequences from all infected beavers (this study), 24 published sequences from wild UK carnivores (11 ferrets, 9 polecats, three mink and one badger), and 38 other published sequences from humans, wild and domestic animals sampled in Europe retrieved from the National Centre for Biotechnology Information (NCBI) database^18^. All sequences were manually curated and aligned in CLC Main Workbench 8.1. A 145 bp alignment was exported to MEGA version 11.0.10 to infer Maximum Likelihood (ML, Hasegawa-Kishino-Yano model), Neighbor Joining (NJ) and Unweighted Pair Group Method with Arithmetic Mean (UPGMA) phylogenies with 1000 bootstrap replications. The optimal model for ML was identified based on minimum Bayesian information criterion (BIC). Single nucleotide polymorphisms (SNPs) and insertion/deletions (indels) were identified.

### Histopathological examination

Samples of brain, heart and liver tissue collected from beaver carcasses during PME were fixed in 10% neutral buffered formalin and processed for haematoxylin and eosin (H&E) staining using standard techniques. Tissue samples were selected for histological assessment of *T. gondii* on the basis of qPCR parasite : host ratios above the 0.001 T*. gondii* genomes per host genome threshold, PCR-RFLP success, and minimal carcass autolysis as reported at post-mortem examination. A total of six tissue samples were examined, along with slides from a seventh individual with PCR values below the selected threshold across all organs for comparison with an apparently low parasite burden. Slides were examined by a board-certified veterinary pathologist for *T. gondii*-like stages (e.g. tachyzoite aggregations, tissue cysts containing bradyzoites) and/or toxoplasmosis hallmarks (e.g. mononuclear cells infiltration, perivascular cuffing, encephalitis, meningitis, gliosis). Samples from two animals with histologic hallmarks of toxoplasmosis were selected for immunohistochemical (IHC) staining for *T. gondii*: one with *T. gondii* detectable by microscopy of H&E stained slides, one not. The original paraffin blocks were processed for IHC and analysed by a commercial provider (University of Liverpool Veterinary Pathology Diagnostic laboratory).

## Supplementary Information

Below is the link to the electronic supplementary material.

Supplementary Material 1

## Data Availability

The 13 SAG3 PCR-RFLP amplicon sequences corresponding to the archetypical ME49 type II lineage (accession numbers PV059145-PV059157) and five genetically distinct SAG3 sequences (accession numbers PV059158-PV059162) obtained from the Eurasian beavers (Castor fiber) are available in the GenBank database. Additional data supporting the findings of this study can be requested from the corresponding author.
