# Supplementary material for: Occurrence and characterisation of Toxoplasma gondii infection in free-living Eurasian beavers, Castor fiber, from southern England
Source: Sci Rep. 2025 Nov 25;15:42516. doi: 10.1038/s41598-025-26504-0 (PMC12660698; doi:10.1038/s41598-025-26504-0)
Supplement: Supplementary file 1 — Supplementary Material 1 [file 41598_2025_26504_MOESM1_ESM.pdf]

Supplementary Table 1. Primers used for PCR-RFLP

Locus	External (5'-3')		Internal (5'-3')		2º amplicon (bp)	Restriction endonuclease	Reference
**5'-SAG2**	5SAG2-Fext<br>5SAG2-Rext	GCTACCTCGAACAGGAACAC<br>GCATCAACAGTCTTCGTTGC	5-SAG2F<br>5-SAG2R	GAAATGTTTCAGGTTGCTGC<br>GCAAGAGCGAACTTGAACAC	242	Sau3AI or MboI	[1], [2]
**3'-SAG2**	3SAG2-Fext<br>3SAG2-Rext	TCTGTTCTCCGAAGTGACTCC<br>TCAAAGCGTGCATTATCGC	3-SAG2F<br>3-SAG2R	ATTCTCATGCCTCCGCTTC<br>AACGTTTCACGAAGGCACAC	222	Hhal	[2], [3]
**SAG3**	P43S1<br>P43AS1	CAACTCTCACCATTCCACCC<br>GCGCGTTGTTAGACAAGACA	P43S2<br>P43AS2	TCTTGTCGGGTGTTCACTCA<br>CACAAGGAGACCGAGAAGGA	226	Ncil	[3]
**BTUB**	Btb(ext)F<br>Btb(ext)R	TCCAAAATGAGAGAAATCGT<br>AAATTGAAATGACGGAAGAA	Btb-F<br>Btb-R	GAGGTCATCTCGGACGAACA<br>TTGTAGGAACACCCGGACGC	411	BsiEI + TaqI	[1], [4]
**GRA6**	GRA6-F1x<br>GRA6-R1	ATTTGTGTTTCCGAGCAGGT<br>GCACCTTCGCTTGTGGTT	GRA6-F1<br>GRA6-R1x	TTTCCGAGCAGGTGACCT<br>TCGCCGAAGAGTTGACATAG	344	Msel	[1], [4]
**Apico**	Apico-Fext<br>Apico-Rext	TGGTTTTAACCCTAGATTGTGG<br>AAACGGAATTAATGAGATTTGAA	Apico-F<br>Apico-R	TGCAAATTCTTGAATTCTCAGTT<br>GGGATTCGAACCCTTGATA	640	AflII + DdeI	[1]

References

[1] Su, C., Zhang, X., Dubey, J.P. Genotyping of *Toxoplasma gondii* by multilocus PCR-RFLP markers: a high resolution and simple method for identification of parasites. *Int. J. Parasitol*. 36, 841–848 (2006).

[2] Howe, D.K., Honore´, S., Derouin, F., Sibley, L.D. Determination of genotypes of *Toxoplasma gondii* strains isolated from patients with toxoplasmosis. *J. Clin. Microbiol.* 35, 1411–1414 (1997).

[3] Grigg, M. E., J. Ganatra, J. C. Boothroyd, and T. P. Margolis. Unusual abundance of atypical strains associated with human ocular toxoplasmosis. *J. Infect. Dis.* 184:633-639 (2001).

[4] Khan, A., Su, C., German, M., Storch, G.A., Clifford, D.B., Sibley, D.L. Genotyping of *Toxoplasma gondii* strains from immunocompromised patients reveals high prevalence of type I strains. *J. Clin. Microbiol.* 43, 5881–5887 (2005).
